# Knowledge, attitude, and practice of pharmacists regarding asthma management: a cross-sectional study in Egypt

**DOI:** 10.1186/s40545-022-00432-0

**Published:** 2022-05-03

**Authors:** Amira S. A. Said, Nadia Hussain, Zelal Kharaba, Amal H. I. Al Haddad, Lamiaa N. Abdelaty, Raghda R. S. Roshdy

**Affiliations:** 1 Department of Clinical Pharmacy, College of Pharmacy, Al Ain University, Al Ain, Abu Dhabi, UAE; 2 AAU Health and Biomedical Research Center (HBRC), Al Ain University, Abu Dhabi, UAE; 3 Department of Clinical Pharmacy, Faculty of Pharmacy, Beni-Suef University, Beni Suef, Egypt; 4 Department of Pharmaceutical Sciences, College of Pharmacy, Al Ain University, Al Ain, Abu Dhabi, UAE; 5 Chief Operations Office, Sheikh Shakbout Medical City (SSMC), Abu Dhabi, UAE; 6 Department of Clinical Pharmacy, Faculty of Pharmacy, October 6 University, Cairo, Egypt; 7 Department of Clinical Pharmacy, Faculty of Pharmacy, Modern University for Technology and Information, Cairo, Egypt

**Keywords:** Asthma, Knowledge, Attitude, Practice, Pharmacists

## Abstract

**Background:**

Asthma is a significant public health issue that poses a substantial health and economic burden. Despite the availability of effective asthma medications, its management remain suboptimal. Recent asthma guidelines have highlighted the importance of pharmacist unique position and its interventional strategies in positively impacting asthma treatment outcomes. Therefore, this study aimed to assess the degree of Egyptian pharmacists’ knowledge, attitudes, as well as their practices towards asthma management in line with the recent asthma guidelines.

**Methods:**

This cross-sectional study was conducted among 800 pharmacists working in different private and governmental sectors. The data were collected using a 37-item pre-validated self-administered KAP questionnaire. The data were analyzed using Student’s t-test and analysis of variance to assess the association between each KAP level and the sociodemographic variables at the significance level of 0.05.

**Results:**

Of the 800 distributed questionnaire, a total of 550 participants (316 Male, and 234 Female) responded, representing a 68.7% response rate. The mean ± SD score of knowledge, attitude, practice, and barrier was 5.49 ± 1.65 (min = 0; max = 8), 23.5 ± 2.84 (min = 15, max = 30), 43.12 ± 8.61 (min = 28, max = 62), and 27.76 ± 3.72 (min = 17, max = 39), respectively. The results showed that poor knowledge, attitude, and practice scores were achieved by 30.54, 0, and 38.72% of participants, respectively.

**Conclusion:**

Our findings revealed the inconsistencies between poor pharmacists’ knowledge and practices with respect to their positive attitudes. The lack of pharmacists’ knowledge and compliance to recent GINA guidelines in this study highlight the crucial need for effective Educational strategies that should better equip pharmacists for their potential role in asthma care.

## Background

Asthma is a chronic inflammatory airway disease that constitutes a prevalent public health problem that affects up to 300 million people worldwide, with an expected increase to 400 million by 2025 [1]. It is estimated that asthma accounts for about one in every 250 global deaths [2]. Although asthma is not curable, its clinical symptoms can be largely controlled with proper management.

Recently, pharmacists’ role has greatly evolved from being medication dispensers to provision of real patient care. Asthma is a typical example of a chronic disease state in which pharmacists are uniquely positioned to provide the needed interventions for a more comprehensive and cost-effective disease management [3].

Despite the continuous advances in asthma treatment and the use of well-established evidence-based guidelines, asthma management remains suboptimal, an issue that raises many public health concerns [4]. This discrepancy between scientific evidence and clinical asthma burden may be due to the disease heterogeneous nature that largely depends on patients’ self-management action plan. The success of patients’ self-management is greatly related to patients’ behaviors as well as the health care professionals’ success to covey proper asthma treatment aspects. Although pharmacists are highly accessible and experienced healthcare professionals, they have been greatly underutilized in asthma care.

For several years, the international guidelines, such as the Global Initiative for Asthma (GINA) and the British Thoracic Society (BTS) Guidelines, have stressed on the importance of self-management as a critical component of asthma care [5]. Several study trials have shown that optimum asthma self-management requires effective pharmacists’ intervention [6, 7].

Since asthma is a chronic cycle of periodic assessment, self-monitoring, and using specialized devices, pharmacists are of high potential to conduct proper asthma education and address patient concerns. Several studies have shown that pharmacists interventions significantly improved asthma symptoms, severity, and quality of life (QOL) and reduced the utilization of healthcare resources [7–9].

Despite that, pharmacists’ interventions in asthma care are highly encouraged in practice, the level of these interventions to be considered minimal or standard are not yet specified. Moreover, the broad variable range of pharmacists’ knowledge, attitudes, and practices in asthma linked to these interventions may not be completely consistent with GINA recommendations.

Previous studies have reported the lack of adherence to asthma international guidelines from both practicing Physicians’ [10] and nurses [11] which motivated us to conduct this study on pharmacists in order to assess their current situation. To our knowledge, this is the first study to assess Egyptian pharmacists’ knowledge, attitude, practice, as well as barriers towards asthma management in line with the recent asthma guidelines.

## Method

### Ethical approval

This study was approved by the Research Ethics Committee of Faculty of Medicine, Beni-Suef University, Egypt. Written informed consent was obtained from all study participants. All further study conduct was in line with the guidelines provided by the Helsinki declaration of 1964 (revised 2013).

### Study design, population, and setting

An observational cross-sectional study was carried out in the cities of Beni-Suef, El Fayoum, and El Minya in Egypt over a period of 8 months from June 2020 to January 2021. An anonymous self-administered questionnaire was prepared to assess pharmacists’ KAP (knowledge attitude, practice) and common barriers towards asthma management in line with the recent GINA guidelines. The questionnaire was adapted and self-designed based on literature review, and aligned with recent GINA asthma guidelines [8, 12]. The guidelines were used to identify all possible aspects of pharmacists’ interventional asthma management. The respondents’ names were not requested for anonymity and to maintain an unbiased response that better reflect the respondents’ opinion. A minimum sample size of 384 pharmacists was calculated using Epi info software with a test power of 80%, confidence interval of 95%, and alpha error of 5%

This KAP questionnaire was conveniently distributed to 800 practicing community and hospital pharmacists working in different private and governmental sectors. Convenience sampling was used in this study for its simplicity, easiness, and for rapid collection of data in a cost-effective way.

The questionnaire was distributed by the researchers who approached each participant to explain the study objectives and obtain their consent. The participating pharmacists were asked to complete the questionnaire and return it to the researcher in a sealed envelope where they were assured that only aggregated data will be reported. Questionnaire completion was estimated to take 15 min, without the consultation of any reference material. The investigator was made available to the participant for any clarification needed.

### Questionnaire development

The final study questionnaire was a 37-item structured self-administered questionnaire that is aimed to assess pharmacists’ KAP (knowledge attitude, practice) and common barriers towards asthma management in line with the recent GINA guidelines.

The questionnaire was validated and examined for content relevance by sending to five professors in pharmacy practice in each of Beni-Suef, El Minya, and Fayoum Universities. Face validity was assessed using convenience sampling of 20 pharmacists who were selected form the three cities and excluded from the main sample study. Participants were asked to evaluate the clarity of the questions and their relevance to the study objectives. The final structure of the questionnaire was amended as required. The Cronbach’s value for internal reliability of individual subscales of the questionnaire after amendments was 0.85, 0.78, 0.81, and 0.83 for knowledge, attitude, practice, and barrier scales, respectively. These values reflect good internal consistency of the questionnaire (> 0.70).

The questionnaire had five sections: the first section (8 questions) collected demographic details; (gender, age, qualification, years of experience, and current area of practice). The second, third, and fourth sections of the questionnaire investigated pharmacists’ knowledge, attitude, and practice using 9, 6, and 14 questions, respectively. The fifth section investigated the encountered barriers during asthma management using 8 questions. Participants were asked to respond to each question in section, three, four, and five by using a 5-point Likert scale (1 = strongly disagree; 2 = disagree; 3 = neutral; 4 = agree; and 5 = strongly agree) for "Ethical approval" and "Study design, population, and setting" Sections and (1 = Always, 2 = Frequently, 3 = Sometimes, 4 = Rarely, 5 = Never) for "Study design, population, and setting" Section and to respond to "Method" Section with yes or no response. The mean weighted response was calculated in the 5-point Likert scale by multiplying the number of respondents in each group by the weight of each response; then all responses were combined into a single composite score (mean weighted response). The mean score was calculated and compared with the hypothesized mean of the Likert scale (midpoint of 3). Despite, the ongoing debate that using the median in Likert scale is a better representative of the central tendency, calculating the mean composite score of ordinal data is an accepted approach in medical research [13].

The total KAP and barrier score were calculated by adding the number of points collected from each section and ranged up to a maximum of 9, 30, 70, and 40 points for the knowledge, attitude, practice, and barriers sections, respectively. Participants were classified according to their scores into 3 categories poor, moderate, and good as follows: < 50% = poor, 50–75% = moderate, and > 75% = good.

### Data analysis

The data were keyed and analyzed in SPSS 23 (IBM Corporation, Chicago, IL, USA). Descriptive statistics were presented using mean and standard deviation for continuous measures, frequencies, and percentages for categorical variables. First, the normality of variables was analyzed using Kolmogorov–Smirnov test; then data were analyzed using Student’s t-test and analysis of variance test to assess the association between each continuous independent variable (KAP scores) and the sociodemographic variables. *P* < 0.05 was considered as a significant association.

## Results

### Demographic characteristics of the participants

Of the 800 distributed questionnaire, a total of 550 participants (316 Male, and 234 Female) responded, representing a 68.7%. response rate. Pharmacists reasons for declining participation were either lack of their interest or time to participate.

The participants’ demographic details are summarized in Table 1. The results showed that the mean ± SD age of pharmacists was 35.31 ± 8.91, with 57.5% male, and 61.2% of participants had bachelor degree. In addition, 30.5% of them had more than 10 years of pharmacy practice experience and 49.6% worked > 40 h/week. Finally, 54.5% were pharmacy owners and 40.5% were working as hospital pharmacists.

**Table1:** Demographic characteristics of recruited participants (*n* = 550)

Characteristic	Category	Number (%)
Gender	Male Female	316 (57.4) 234 (42.5)
Age (yr)	< 35 36–34 > 47	325 (59%) 135 (24.5%) 90 (16.3)
Education Level	BS Pharmacy Pharm. D Master Ph.D.	340 (61.8) 80 (14.5) 70 (12.7) 60 (10.9)
Pharmacy Type	Hospital pharmacy Community pharmacy	223 (40.5) 327 (59.4)
Years of pharmacy practice experience (yr)	< 5 6–10 > 10	150 (27.3) 232 (42.1) 168 (30.5)
Pharmacy position	Owner Staff	300 (54.5) 250 (45.4)
Weekly working hours	1–20 h 20–40 h > 40 h	67 (12.2) 210 (38.2) 273 (49.6)
Number of Daily patients	< 10 10–50 50–100 > 100	55 (10) 212 (38.5) 224 (40.7) 56 (10.2)

### Description of the KAP scores

**Fig. 1:**
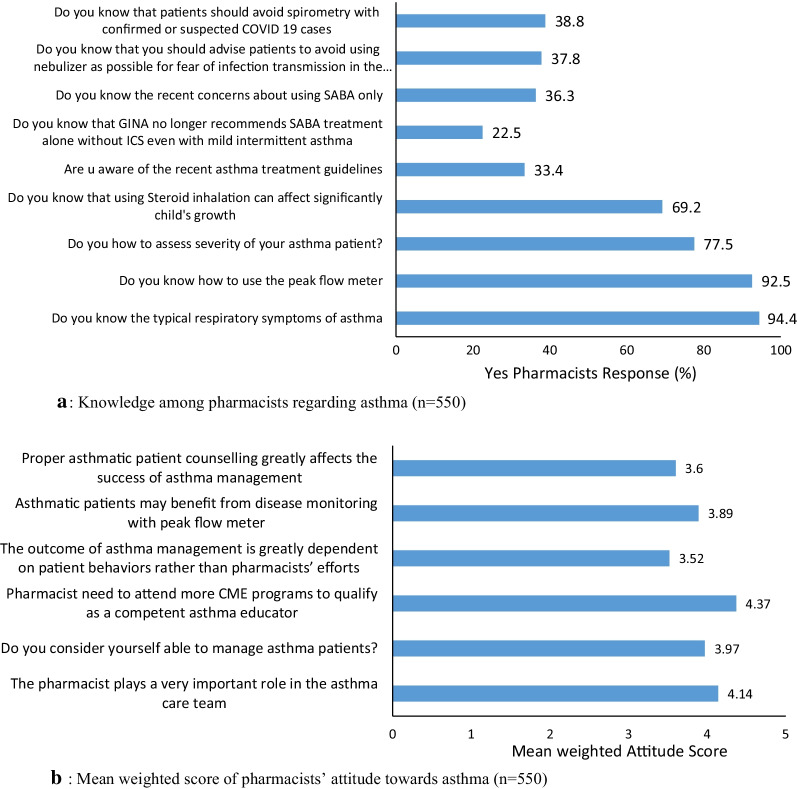

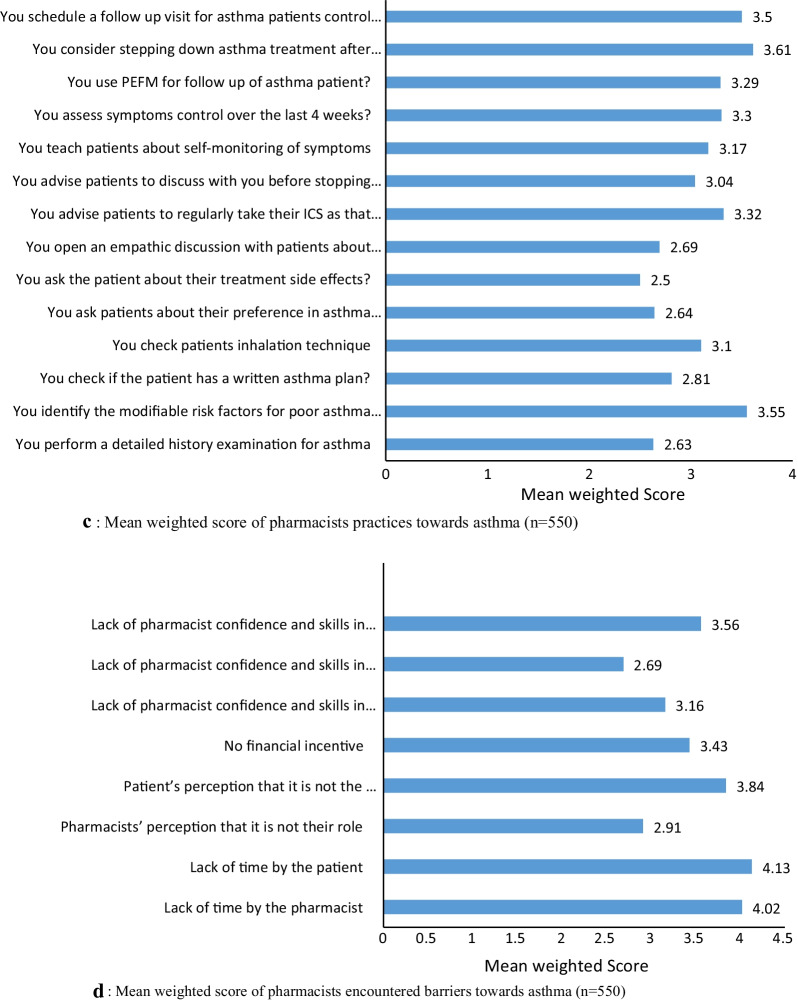
**a** Knowledge among pharmacists regarding asthma (*n* = 550). **b** Mean weighted score of pharmacists’ attitude towards asthma (*n* = 550). **c** Mean weighted score of pharmacists practices towards asthma (*n* = 550). **d** Mean weighted score of pharmacists encountered barriers towards asthma (*n* = 550).

The responses of pharmacists and the reliability analysis on various statements regarding KAP and barriers towards asthma management are illustrated in Table 2 and Fig. 1a–d. The Cronbach’s alpha coefficient for knowledge, attitude, practice, and barrier variable was calculated and found to be greater than 0.7 for all, which means good reliability. [8, 12].

**Table 2:** The responses of pharmacists and the reliability analysis on various statements regarding KAP and barriers towards asthma management (n = 550)

**Questions**			Yes, Response (%) or (Mean ± SD)	Cronbach’s alpha
Knowledge	1.	Do you know the typical respiratory symptoms of asthma	522 (94.4)	0.85
	2.	Do you know how to use the peak flow meter	509 (92.5)	
	3.	Do you how to assess severity of your asthma patient?	426 (77.5)	
	4.	Do you know that using Steroid inhalation can affect significantly child's growth	381 (69.2)	
	5.	Are you aware of the recent asthma treatment guidelines	183 (33.4)	
	6.	Do you know that GINA no longer recommends SABA treatment alone without ICS even with mild intermittent asthma	124 (22.5)	
	7.	Do you know the recent concerns about using SABA only	200 (36.3)	
	8.	Do you know that you should advise patients to avoid using nebulizer as possible for fear of infection transmission in the current epidemic situation	208 (37.8)	
	9.	Do you know that patients should avoid spirometry with confirmed or suspected COVID-19 cases	210 (38.8)	
Attitude	1.	The pharmacist plays a very important role in the asthma care team	4.14 ± 1.10	0.78
	2.	Do you consider yourself able to manage asthma patients?	3.97 ± 1.07	
	3.	Pharmacist need to attend more CME programs to qualify as a competent asthma educator	4.37 ± 0.94	
	4.	The outcome of asthma management is greatly dependent on patient behaviors rather than pharmacists’ efforts	3.52 ± 1.15	
	5.	Asthmatic patients may benefit from disease monitoring with peak flow meter	3.89 ± 1.9	
	6.	Proper asthmatic patient counseling greatly affects the success of asthma management	3.60 ± 1.13	
Practice	1.	Do you perform a detailed history examination for asthma	2.63 ± 0.97	0.81
	2.	Do you identify the modifiable risk factors for poor asthma outcomes?	3.55 ± 1.32	
	3.	Do you check if the patient has a written asthma plan?	2.81 ± 1.16	
	4.	Do you check patients inhalation technique	3.10 ± 0.77	
	5.	Do you ask patients about their preference in asthma treatment	2.64 ± 1.36	
	6.	Do you ask the patient about their treatment side effects?	2.50 ± 0.88	
	7.	Do you open an empathic discussion with patients about their adherence	2.69 ± 0.97	
	8.	Do you advise patients to regularly take their ICS as that might worsen their asthma medications	3.32 ± 1.22	
	9.	Do you advise patients to discuss with you before stopping any of their medication	3.04 ± 0.91	
	10.	Do you teach patients about self-monitoring of symptoms	3.17 ± 1.56	
	11.	Do you assess symptoms control over the last 4 weeks?	3.30 ± 0.59	
	12.	Do you use PEFM for follow-up of asthma patient?	3.29 ± 0.92	
	13.	Do you consider stepping down asthma treatment after proper asthma	3.61 ± 1.11	
	14.	Do you schedule a follow-up visit for asthma patients control for 3 months	3.50 ± 1.21	
Barriers	1.	Lack of time by the pharmacist	4.02 ± 2.1	0.83
	2.	Lack of time by the patient	4.13 ± 0.84	
	3.	Pharmacists’ perception that it is not their role	2.91 ± 0.92	
	4.	Patient’s perception that it is not the pharmacist’s role	3.84 ± 0.69	
	5.	No financial incentive	3.43 ± 0.92	
	6.	Lack of pharmacist confidence and skills in asthma management	3.16 ± 0.88	
	7.	Lack of pharmacist confidence and skills in asthma counseling	2.69 ± 0.59	
	8.	Lack of pharmacist confidence and skills in asthma monitoring	3.56 ± 1.10

### Knowledge score

The mean knowledge score was 5.49 ± 1.65 (median = 5; minimum = 0; maximum = 8). When dividing the score into three categories, the results showed that 168 (30.54%) had poor knowledge (scores < 5), 225 (40.9%) had moderate knowledge (scores between 5 and 6), and 157 (28.5%) had good knowledge (scores of 7 and above). As shown in Table 3, the results of the bivariate analysis showed that a significantly higher mean knowledge score was found for hospital pharmacists (5.69) compared to community pharmacists (5.01). In addition, pharmacists with longer experience years (> 10 yr) showed better knowledge score (6.35) compared to lesser years of experience: 6–10 yr (5.61) and < 5 (5.53).

**Table 3:** Bivariate analysis of factors associated with the knowledge, attitude, and practice scores

Variable	Knowledge (Mean ± SD)	Attitude (Mean ± SD)	Practice (Mean ± SD)	Barriers (Mean ± SD)
Gender				
Male	5.69 ± 2.31	22.69 ± 3.15	45.86 ± 5.94	30.62 ± 3.69
Female	5.48 ± 1.12	23.97 ± 3.25	45.97 ± 5.36	29.82 ± 4.85
*p*-value	0.31	0.07*	0.23	0.07
Age (yr)				
< 35	5.48 ± 1.01	23.74 ± 2.15	44.16 ± 5.47	32.21 ± 4.32
36–34	5.51 ± 0.75	24.11 ± 3.01	43.28 ± 4.97	32.11 ± 5.11
> 47	5.68 ± 1.1	23.58 ± 1.69	42.91 ± 4.25	32.91 ± 4.23
* p*-value	0.33	0.6	0.09	0.07
Education level				
BS Pharmacy	5.48 ± 1.25	23.45 ± 4.02	43.71 ± 5.14	34.45 ± 3.36
Pharm. D	5.55 ± 1.36	23.83 ± 3.17	45.67 ± 5.55	35.15 ± 3.25
Master	5.71 ± 1.04	23.86 ± 4.01	44.28 ± 6.15	34.66 ± 3.15
Ph.D.	5.23 ± 0.98	23.65 ± 4.15	43.61 ± 5.97	34.15 ± 3.66
*p*-value	0.7	0.41	0.06	0.09
Pharmacist				
Hospital pharmacist	5.69 ± 0.36	23.91 ± 1.54	44.91 ± 3.85	31.52 ± 3.17
Community pharmacist	5.01 ± 0.48	23.69 ± 2.51	41.11 ± 4.87	30.31 ± 3.74
*p*-value	< 0.001*	0.27 ± 1.25	< 0.001*	0.008
Years of experience (yr)				
< 5	5.53 ± 1.25	23.26 ± 2.36	41.21 ± 4.85	32.62 ± 3.17
6–10	5.61 ± 1.01	23.58 ± 3.01	43.14 ± 5.94	32.22 ± 3.15
> 10	6.35 ± 1.61	23.81 ± 3.11	44.28 ± 3.69	33.11 ± 3.66
*p*-value	0.039*	0.68	< 0.001*	0.08
Pharmacy position				
Owner	5.97 ± 2.15	23.67 ± 4.05	41.39 ± 5.13	31.86 ± 2.97
Staff	5.69 ± 1.02	23.47 ± 3.94	41.69 ± 5.19	31.42 ± 3.48
*p*-value	0.41	0.25	0.62	0.117
Weekly working hours				
1–20 h	5.33 ± 0.15	23.45 ± 4.15	42.16 ± 5.47	32.84 ± 3.15
20–40 h	5.74 ± 0.95	23.83 ± 3.25	42.11 ± 5.17	32.90 ± 4.01
> 40 h	5.67 ± 0.36	23.65 ± 3.74	42.66 ± 5.36	32.81 ± 3.69
*p*-value	0.3	0.5	0.1	0.9
Number of Daily patients				
< 10	5.19 ± 0.94	22.82 ± 1.64	43.71 ± 5.17	31.45 ± 3.97
10–50	5.61 ± 0.47	22.49 ± 3.62	43.28 ± 6.11	32.91 ± 1.69
50–100	5.39 ± 0.25	22.46 ± 2.58	43.69 ± 4.97	33.14 ± 2.87
> 100	5.75 ± 1.20	22.63 ± 4.15	44.13 ± 5.28	30.25 ± 3.84
*p*-value	0.37	0.5	0.049*	0.02

In addition, the Pearson correlation tests showed a positive and significant correlation amid KAP scores as follows: knowledge–attitude (*r* = 0.294, *p* < 0.001), attitude–practice (*r* = 0.211, *p* < 0.001), and knowledge–practice (*r* = 0.234, *p* < 0.001)

*p < 0.05

### Attitude score

The mean attitude score was 23.5 ± 2.84 (median = 24; minimum = 15; maximum = 30). When dividing the score into three categories, the results showed that 0 (0%) had poor attitude (scores < 15), 96 (17.4%) had moderate attitude (scores between 15 and 21), and 441 (80.2%) had good attitude (> 21). As shown in Table 3, a significantly higher attitude score was found in females compared to males (23.97 vs 22.69).

### Practice score

The mean practice score was 43.12 ± 8.61 (median = 45.5; minimum = 28; maximum = 62). When dividing the score into three categories, the results showed that 213 (38.72%) had poor practice (scores < 35), 259 (47.1%) had moderate practice (scores between 35 and 52), and 75 (13.6%) had good practice (> 52). As shown in Table 3, a significantly higher mean practice score was found in hospital pharmacists compared to community pharmacists (44.91 vs 41.11), pharmacists with longer years of experience (44.28), and in pharmacists with > 100 daily patients (44.13), compared to other categories.

### Barrier score

The mean barrier score was 27.76 ± 3.72 (median = 28; minimum = 17; maximum = 39). When dividing the score into three categories, the results showed that 15 (2.72%) showed poor barrier score (scores < 20), 398 (72.36%) had moderate barrier score (scores between 20 and 30), and 137 (24.9%) showed good barrier scores (> 30).

**Fig. 2:**
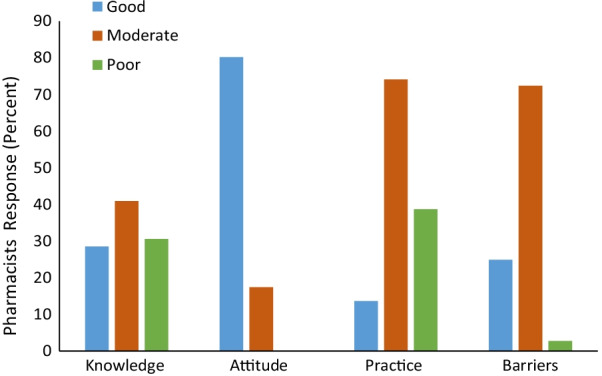
Mean knowledge, attitudes, practices, and barrier scores of study pharmacists. Knowledge score < 5 = poor knowledge, 5–6 score = moderate knowledge, and ≥ 7 score = good knowledge. Attitude score < 15 = poor attitude, 15–21 score = moderate attitude, and > 21 score = good attitude. Practice score < 35 = poor practice, 35–52 score = moderate practice, and > 52 score = good practice. Barrier score < 20 = poor barrier, 20–30 score = moderate barrier, and > 30 = good barrier

The knowledge, attitudes, practices, and barrier scores of study pharmacists are illustrated in Fig. 2.

## Discussion

Despite the availability of effective asthma treatments and updated international guidelines, many asthmatic patients remain largely uncontrolled according to Global Initiative for Asthma (GINA) criteria [14].

Pharmacists drug expertise and their easy accessibility to patients often represent an underutilized resource for proper asthma management. Therefore, current national and international guidelines continually endorse pharmacists’ promising role for the provision of ongoing asthma care. However, pharmacists specific interventional role in these guidelines is yet not clearly articulated [8]. Moreover, their provided quality of asthma care greatly depends on their interventional knowledge, attitudes, and practices which may not be completely consistent with GINA recommendations [15].

Therefore, this study aimed to investigate pharmacists’ knowledge, attitudes, practices, and perceived barriers towards asthma management in Egypt, based on current asthma management guidelines.

The results of this study showed that less than half of the pharmacists (28.5%) who had good asthma knowledge with a significant higher knowledge score were reported for hospital pharmacists Vs community pharmacists and for pharmacists with longer experience years. A good knowledge score was reported for knowing asthma symptoms (94.4%), how to use the PFM (92%), and assessing asthma severity (77.5%).

This finding of poor pharmacists’ knowledge was in agreement with other previous studies conducted in France [16], Qatar [17], and Saudi Arabia [18]. Consistently, a previous study from Pakistan reported that community pharmacists failed to acknowledge most aspects of the primary asthma signs, its triggers, or correct inhalers use. Moreover, a previous study among Nigerian community pharmacists reported that only 34.8% and 11.2% of pharmacists had good knowledge and demonstrated good practice with GINA reports, respectively [19]. Another study among Turkish pharmacists’ reported them to have insufficient or incorrect asthma knowledge and further suggested adopting different educational methods to correct asthma misconceptions [20]. In addition, a majority of Sudanese pharmacist in another study lacked the basic knowledge to properly educate their asthma patients about their disease or correct inhalers use [21].

GINA guidelines are considered the gold standard for asthma diagnosis and management. Several previous studies have strongly correlated high level of asthma practice with good knowledge of asthma guidelines [12, 22]. Using guidelines in practice was reported to minimize treatment inconsistencies and reduce avoidable hospitalizations and costs [23].

It is of note in this study that only 33.3% of pharmacists were aware of the most updated GINA guidelines. This is consistent with several previous studies that either reported lack of pharmacists knowledge [10, 12] or compliance with asthma clinical guidelines in practice [22].

Recently, GINA 2020 guidelines have published a drastic change in step 1 mild asthma management. Based on scientific evidence GINA no longer supports short acting beta agonist (SABA) monotherapy for mild asthmatic patients, an approach that has been used for the last 30 years. The current evidence recommends receiving symptom driven (mild asthma) or daily inhaled corticosteroids (ICS) to reduce exacerbations risk. However, in this study, pharmacists were not aware about GINA recent SABA concerns (63.7%) or that GINA no longer recommends SABA alone with ICS even in mild intermittent asthma (77.5%). Indeed, regularly updating healthcare professionals (HCPs) and especially pharmacists with recent guidelines will be extremely valuable at all levels to easily apply the evidence-based strategies for better patient care.

Currently, the worldwide COVID-19 outbreak is highly challenging in asthma patients’ management: First, the dilemma of the possible substantial overlaps between the clinical presentation of uncontrolled asthma and COVID-19; second the possibility of spreading COVID-19 easily through asthma drug aerosols. In that context, GINA guidelines now recommend not using any aerosolization procedure, such as nebulization, spirometry or peak expiratory flow meter (PEFM) for asthmatic patients with suspected or confirmed COVID-19 due to the potential risk of its transmission [25].

This study showed that only 38.3% of pharmacists knew that they should avoid spirometry with confirmed or suspected COVID 19 cases. This is somehow worrisome, as insufficient pharmacist knowledge about this important update in guidelines jeopardizes many vulnerable patients for COVID-19 transmission, taking into consideration that aerosol droplets can remain for hours in the air [23].

This poor knowledge about the current GINA guidelines in this study reflects the importance of conducting educational asthma programs that should keep pharmacists with the needed up to date knowledge. Indeed, a well-informed pharmacist who comply with the clinical guidelines should be able to make proper therapeutic decisions for more effective asthma management [10, 12]. In order to design better educational strategies for pharmacists, their understanding of the relevant guidelines should be regularly assessed.

When the goal is to change clinical practice, targeting attitude and knowledge are equally important as without the proper pharmacist attitude, his knowledge will not be properly applied. Optimistically, a majority of pharmacists in this study had good attitude towards asthma management (80.2%) with female respondents expressed significantly higher positive attitude compared to males. Several previous studies have reported that improved pharmacist knowledge and attitude are a prerequisite to effective asthma care where patients are more empowered to be effective contributor in their disease management [7, 9, 26].

Overall pharmacists in this study showed high mean weighted response towards needing educational programs (4.37) and understanding their important role in asthma care (4.14). Such positive attitude is highly encouraging and consistent with a previous Turkish study that reported that 80% of the 52% pharmacists with poor knowledge reported the need for further education. Another Finnish study reported that 40% of pharmacist had poor knowledge, yet > 80% believed their important role in practice [16].

In addition, several previous studies have demonstrated significant improvements in knowledge and attitude among pharmacists who attended the educational programs [27]. Maintaining continuous asthma education approach would help ensure compliance with proper asthma care practice [28].

The good pharmacists practice in this study was illustrated by only 13.6% of studied pharmacists. Noteworthy in this study, hospital pharmacists showed significantly higher knowledge and better practice, compared to community pharmacists. These results were not surprising as hospital pharmacists are more existent active partners in clinical decision processes compared to community pharmacists. Other previous studies have also reported better knowledge and attitudes of hospital pharmacists vs community pharmacist in asthma care [29].

It is well known that PEFM is one of the commonest methods used for asthma initial evaluation and monitoring. Although, in our study, 92.5% reported knowing how to use the PEFM, the mean weighted score for using a PEFM for asthma patient follow-up was not as high (3.29). This is consistent with other previous studies that also reported poor PEFR use in patients follow-up [8]. Another previous study among Egyptian physicians’ showed that only 22.5% used PEFM for asthmatic patients follow-up [10]. Evidence has shown that patients with written asthma action plans that include PEFM assessment based on previous personal best readings consistently improved asthma outcome [30].

As noted, the lowest mean weighted score for pharmacists practice in this study was for asking patients about treatment side effects (2.5), discussing patients’ adherence (2.69), checking if patients have a written asthma plan (2.81), performing a detailed asthma history examination (2.64), or asking patients about their preference in asthma treatment (2.63). The episodic and chronic nature of asthma disease, which make pharmacists interventions in designing patients self-management plans, are a highly needed for sustained asthma control. Such poor pharmacists practice in guiding patients towards asthma self-management in this study needs urgent solutions.

It has been previously reported that pharmacists self-management education significantly improved asthma patients’ knowledge, attitudes, and adherence which translated to better asthma control [31]. Another previous study has reported that pharmacist education about adherence was identified as much better predictors of adherence than neither socioeconomic nor clinical factors [31]. In addition, several studies have correlated asthma fatality with insufficient asthma knowledge, noncompliance, and improper management [2]. Studies have persistently shown that educating asthma patients about self-management and using PFM significantly contributed to better QOL and asthma control [32]. Several studies in literature have correlated poor levels of knowledge and practice among pharmacists as barriers to effective asthma care [1, 33].

In this study, pharmacists identified most important and common barriers to providing asthma care. For pharmacist-related factors, the highest mean weighted score was for barriers, such as lack of pharmacist time (4.13), lack of pharmacist confidence in skills in asthma monitoring (3.56), management (3.16), counseling (2.69), and besides lack of financial incentive (3.43). Similarly, other previous studies have consistently reported lack of pharmacists time and education as major barriers to provision of asthma care services [3]. For patient-related factors the highest mean weighted score was for lack of patient time (4.13) plus patient perception that it is not the pharmacist role (3.84). Similar types of barriers were also identified in several previous studies [3, 34]. Understanding and addressing these barriers are of prime importance in developing tailored intervention asthma programs that achieve the desirable optimum asthma care.

The present study has several strengths and limitations. First, a relatively small sample size was studied, a single country, and therefore, the results cannot be generalized to other developing countries with different populations and economic conditions. Despite these limitations, we believe that this study was the first study to acknowledge pharmacists’ knowledge, attitude, practice, and barriers towards asthma management and their compliance with international GINA guidelines. The results of the study were helpful to understand the discrepancies between pharmacists’ good attitude and poor knowledge and practice. This study stressed on the importance of conducting asthma education programs to improve pharmacists’ knowledge that should translate to better practice. However, the real impact of these education programs on improving pharmacist practice and asthma outcomes still needs to be investigated.

## Conclusion

Asthma in Egypt is still a well-neglected entity unlike other chronic diseases, like diabetes mellitus and hypertension. Overall, our results have shown pharmacists’ inadequate knowledge and practices regarding asthma which does not comply with the current GINA guidelines. Optimistically, a majority of pharmacists in this study had good attitude towards asthma management and identified their need for educational programs for more effective asthma care. Continuous medical education about current clinical asthma guidelines could be an effective future approach to narrow the gap between the recommended and the actual asthma management practices. In addition, this study showed that the lack of pharmacist confidence in managing asthma and lack of time were major barriers to provision of proper asthma care. Recognizing the specific areas of deficits in asthma care is highly important in devising better asthma prevention and management strategies.

## Data Availability

The datasets used and/or analyzed during the current study are the corresponding author on reasonable request.
