# Correction to: Knowledge, attitude, and practice of pharmacists regarding asthma management: a cross-sectional study in Egypt

**DOI:** 10.1186/s40545-022-00436-w

**Published:** 2022-05-23

**Authors:** Amira S. A. Said, Nadia Hussain, Zelal Kharaba, Amal H. I. Al Haddad, Lamiaa N. Abdelaty, Raghda R. S. Hussein

**Affiliations:** 1 Department of Clinical Pharmacy, College of Pharmacy, Al Ain University, Al Ain, Abu Dhabi, UAE; 2 AAU Health and Biomedical Research Center (HBRC), Al Ain University, Abu Dhabi, UAE; 3 Department of Clinical Pharmacy, Faculty of Pharmacy, Beni-Suef University, Beni Suef, Egypt; 4 Department of Pharmaceutical Sciences, College of Pharmacy, Al Ain University, Al Ain, Abu Dhabi, UAE; 5 Chief Operations Office, Sheikh Shakbout Medical City (SSMC), Abu Dhabi, UAE; 6 Department of Clinical Pharmacy, Faculty of Pharmacy, October 6 University, Cairo, Egypt; 7 Department of Clinical Pharmacy, Faculty of Pharmacy, Modern University for Technology and Information, Cairo, Egypt

## Correction to: Journal of Pharmaceutical Policy and Practice (2022) 15:35 https://doi.org/10.1186/s40545-022-00432-0

Following the publication of the original article [1], we were notified that the last author’s last name was incorrect.

Originally published name: Raghda R. S. Roshdy

Correct name: Raghda R. S. Hussein

The original article has been corrected.
